# Power of multifactor dimensionality reduction and penalized logistic regression for detecting gene-gene Interaction in a case-control study

**DOI:** 10.1186/1471-2350-10-127

**Published:** 2009-12-04

**Authors:** Hua He, William S Oetting, Marcia J Brott, Saonli Basu

**Affiliations:** 1 Division of Biostatistics, School of Public Health, University of Minnesota, Minnesota, USA; 2 Department of experimental and clinical pharmacology, College of Pharmacy and Institute of Human Genetics, University of Minnesota, Minnesota, USA

## Abstract

**Background:**

There is a growing awareness that interaction between multiple genes play an important role in the risk of common, complex multi-factorial diseases. Many common diseases are affected by certain genotype combinations (associated with some genes and their interactions). The identification and characterization of these susceptibility genes and gene-gene interaction have been limited by small sample size and large number of potential interactions between genes. Several methods have been proposed to detect gene-gene interaction in a case control study. The penalized logistic regression (PLR), a variant of logistic regression with *L*_2 _regularization, is a parametric approach to detect gene-gene interaction. On the other hand, the Multifactor Dimensionality Reduction (MDR) is a nonparametric and genetic model-free approach to detect genotype combinations associated with disease risk.

**Methods:**

We compared the power of MDR and PLR for detecting two-way and three-way interactions in a case-control study through extensive simulations. We generated several interaction models with different magnitudes of interaction effect. For each model, we simulated 100 datasets, each with 200 cases and 200 controls and 20 SNPs. We considered a wide variety of models such as models with just main effects, models with only interaction effects or models with both main and interaction effects. We also compared the performance of MDR and PLR to detect gene-gene interaction associated with acute rejection(AR) in kidney transplant patients.

**Results:**

In this paper, we have studied the power of MDR and PLR for detecting gene-gene interaction in a case-control study through extensive simulation. We have compared their performances for different two-way and three-way interaction models. We have studied the effect of different allele frequencies on these methods. We have also implemented their performance on a real dataset. As expected, none of these methods were consistently better for all data scenarios, but, generally MDR outperformed PLR for more complex models. The ROC analysis on the real dataset suggests that MDR outperforms PLR in detecting gene-gene interaction on the real dataset.

**Conclusion:**

As one might expect, the relative success of each method is context dependent. This study demonstrates the strengths and weaknesses of the methods to detect gene-gene interaction.

## Background

Genetic mapping of a trait involves implementation of a number of statistical strategies to identify relative position(s) of gene(s) influencing the trait in the genome. Such strategies have been a major breakthrough in identification of genes responsible for simple human diseases or traits. More than 1600 genes have been identified [1] for simple human traits such as nail patella syndrome [2], cystic fibrosis [3] since the 1980s. Many complex traits of medical relevance such as Diabetes, Asthma, and Alzheimer's disease are controlled by multiple genes. Interaction between genes, low penetrance, and environmental factors make the gene discovery difficult for these complex traits. A common study design for genetic mapping of a trait is a case-control study design where genotype data on a large number of single nucleotide polymorphisms (SNPs) are collected for a number of cases and controls to study the association between these SNPs and the trait. There is a growing evidence that these SNPs interact with each other in determining the susceptibility to complex traits or diseases. The investigation of such gene-gene interactions presents new statistical challenges as the number of potential interactions between the SNPs can be large.

Gene-gene interaction or epistatis has been defined in multiple different ways [4-6]. Biological epistasis, as defined by [7], is the physical interactions among biomolecules in gene regulatory networks and biochemical pathways at the cellular level of an individual. Statistical epistasis, as defined by [8], is the deviation from additivity in a linear mathematical model that describes the relationship between the genotypes and phenotype at a population level. The relationship between biological and statistical epistasis is often confusing. It is important to understand the relationship if we make biological inferences from statistical results [9]. The focus of the present study is the detection and characterization of statistical epistasis in human populations.

The traditional parametric statistical approach to modeling the relationship between disease status and SNPs is logistic regression which has some obvious limitations. As each additional main effect is included in the logistic model, the number of possible interaction terms grow exponentially. Due to the sparseness of the data in high dimensions, parameter estimates often tend to have large standard errors, making it difficult to detect interaction. Several methods have been proposed to detect gene-gene interaction in a case control study design.

These methods can be categorized broadly into parametric and nonparametric approaches. Parametric methods assume a model to describe the effect of each SNP or combination of SNPs on the disease. Some examples of parametric approaches are ridge penalized logistic regression [10], LASSO [11], and logic regression [12]. All these methods try to address the potential problem of fitting gene-gene interaction models through traditional logistic regression and propose alternative ways to identify interactions. Recently, [10] proposed the penalized logistic regression (PLR), which is analogous to ridge regression to detect gene-gene interaction. Under certain models as well as on two real datasets, they demonstrated that PLR outperforms Multifactor Dimensionality Reduction method [13] and Flextree [14]. The use of penalized logistic regression [10] showed substantial promise for overcoming the limitations of traditional logistic regression.

The nonparametric approaches for detection gene-gene interaction search through different levels of interaction regardless of the significance of the main effects. In nonparametric methods, several data mining approaches have been developed and employed in the detection of gene-gene interactions. Some of these approaches are Combinatorial Partitioning Method or CPM [15], Neural Network [16], Multifactor Dimensionality Reduction or MDR [13,17]. These methods detect the relevant interactions between the SNPs by either reducing the dimension of the vast genetic data or recognizing the useful hidden patterns. These approaches do not make any assumption about the nature of dependence between the trait and the SNPs; instead, it is determined from the data. This makes the nonparametric methods more flexible compared to the parametric methods. Recently [18] has compared a number of different models and MDR came out to be a performing fairly well across all comparison. Hence, we have selected MDR as the representative of the nonparametric approaches and compared its performance with PLR through extensive simulations.

In this paper, we have studied the power of MDR and PLR for detecting gene-gene interaction in a case-control study for various interaction models. As compared to [10], we have considered a wide variety of 2-way and 3-way interaction models with strong or weak effects of the predictors. We have also studied the influence of the minor allele frequencies of the SNPs on the performance of the methods. Our findings were somewhat different from [10]. We did not find PLR consistently outperforming MDR. If there was an additive sub-model that explained the interaction among the SNPs, PLR performed better. On the other hand, MDR was particularly good in detecting the weak effects of a purely epistatic interaction. The prediction errors were generally lower for MDR as compared to PLR. Moreover, the ROC analysis on the real dataset suggested that MDR outperformed PLR in detecting gene-gene interaction on the real dataset.

## Results and Discussion

### Simulation 1 & Results

We have compared the power of MDR and PLR for detecting 2-way interaction in a case-control study through extensive simulations. We generated 9 models with different magnitudes of interaction effect. For each model, we simulated 100 datasets. Each dataset contained 400 samples (200 cases and 200 controls) and 20 SNPs, only 2 of which (SNP1 and SNP2) were associated with the disease. We considered a wide variety of models such as models with just main effects, models with only 2nd-order interaction effects or models with both main and interaction effects to represent the underlying genetic models for the effect of the associated SNPs on the disease. The coefficients of the models were selected somewhat arbitrarily to cover a variety of prevalence and heritability of the disease. If we denote the alleles of SNP1, SNP2 by (A, a) and (B, b) respectively, then the 9 models used in the simulation were

1. Model 1: Logit(p) = -6.9 + 7.31 I(SNP1 = Aa, SNP2 = BB) + 7.31 I(SNP1 = AA, SNP2 = Bb) + 7.31 I(SNP1 = AA, SNP2 = BB)

2. Model 2: Logit(p) = -6.9 + 6.5 I(SNP1 = AA) + 6.5 I(SNP2 = BB) - 13 I(SNP1 = AA, SNP2 = BB)

3. Model 3: Logit(p) = -6.9 + 6.9 I(SNP1 = AA) + 6.9 I(SNP2 = BB)

4. Model 4: Logit(p) = -2.197 + 0.811 I(SNP1 = AA) + 0.811 I(SNP2 = BB)

5. Model 5: Logit(p) = -2.197 + 0.811 I(SNP1 = AA) + 0.811 I(SNP2 = BB) + 1.96 I(SNP1 = AA, SNP2 = BB)

6. Model 6: Logit(p) = -2.197 + 0.811 I(SNP1 = AA) + 0.811 I(SNP2 = BB) - 1.622 I(SNP1 = AA, SNP2 = BB)

7. Model 7: Logit(p) = -2 + 1 I(SNP1 = AA) + 1 I(SNP2 = BB) - 2 I(SNP1 = AA, SNP2 = BB)

8. Model 8: Logit(p) = -2 + 1 I(SNP1 = Aa) + 1 I(SNP2 = Bb) - 2 I(SNP1 = Aa, SNP2 = Bb)

9. Model 9: Logit(p) = -0.575 + 1 I(SNP1 = Aa, SNP2 = Bb) + 1 I(SNP1 = Aa, SNP2 = BB) - 1 I(SNP1 = AA, SNP2 = Bb).

**Figure 1:**
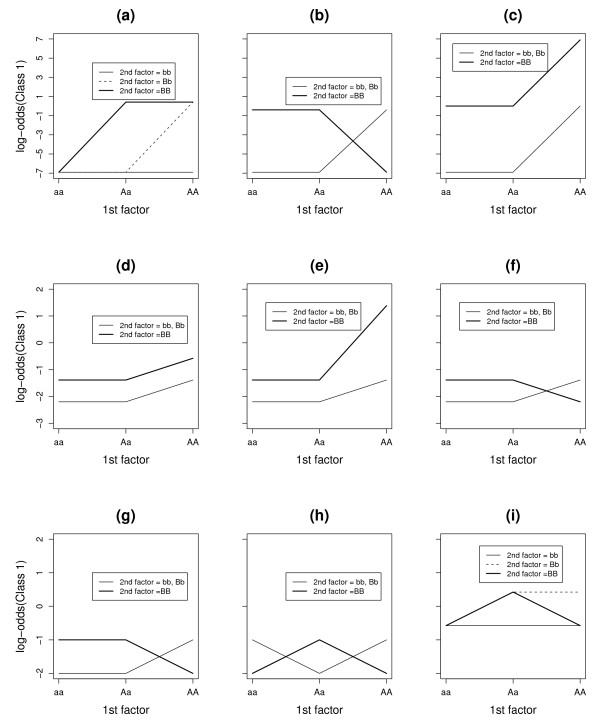
**The pattern of log-odds for 2-way interaction models**. The patterns of log-odds for class1(affected) for different levels of the two SNPs (SNP1 and SNP2) under nine different models with 2-way interactions. The subfigures (a), (b), (c), (d), (e), (f), (g), (h), (i) represent Model 1, Model 2, Model 3, Model 4, Model 5, Model 6, Model 7, Model 8 and Model 9 (Table 1) respectively.

Three levels of the 2 associated SNPs were distributed so that the conditional probabilities of being diseased were as in Table 1 (often referred as the penetrance tables). The levels of the remaining 18 SNPs were in Hardy-Weinberg equilibrium separately for cases and controls. Figure 1 shows the log odds of being diseased for all possible combinations of levels of the 2 associated SNPs. For Model 2 to Model 8, the SNPs had main effects, while for Model 1 and Model 9, they did not have any main effect. The log odds were additive for Model 3, 4, while the other 7 models show certain interaction effects. We simulated all the models under different allele frequencies such as 0.1, 0.2 and 0.5 to assess the effect of allele frequency on the power of each approach. The heritability with the minor allele frequency of 0.50 for the causal SNPs were 51%, 29%, 50%, 4%, 19%, 2%, 4%, 3.5% and 6% for Model 1, 2, 3, 4, 5, 6, 7, 8 and 9 respectively. The prevalence of the disease was 0.18, 0.15, 0.25, 0.15, 0.18, 0.14, 0.18, 0.19, and 0.48 for Model 1, 2, 3, 4, 5, 6, 7, 8 and 9 respectively.

**Table 1:** Penetrance Tables for 2-way interactions. Penetrance tables of nine different models with 2-way interactions. SNP1 has three genotypes AA, Aa, aa and SNP2 has three different genotypes BB, Bb, bb. Each cell of a penetrance table represents the probability of being affected given the cell genotype.

**Model 1**				**Model 2**				**Model 3**			
		
	**bb**	**Bb**	**BB**		**bb**	**Bb**	**BB**		**bb**	**Bb**	**BB**
		
aa	0	0	0	aa	0	0	.4	aa	0	0	.5
		
Aa	0	0	.6	Aa	0	0	.4	Aa	0	0	.5
		
AA	0	.6	.6	AA	.4	.4	0	AA	.5	.5	1
		
**Model 4**				**Model 5**				**Model 6**			
		
	**bb**	**Bb**	**BB**		**bb**	**Bb**	**BB**		**bb**	**Bb**	**BB**
		
aa	.1	.1	.2	aa	.1	.1	.2	aa	.1	.1	.2
		
Aa	.1	.1	.2	Aa	.1	.1	.2	Aa	.1	.1	.2
		
AA	.2	.2	.36	AA	.2	.2	.8	AA	.2	.2	.1
		
**Model 7**				**Model 8**				**Model 9**			
		
	**bb**	**Bb**	**BB**		**bb**	**Bb**	**BB**		**bb**	**Bb**	**BB**
		
aa	.12	.12	.27	aa	.12	.27	.12	aa	.36	.36	.36
		
Aa	.12	.12	.27	Aa	.27	.12	.27	Aa	.36	.60	.60
		
AA	.27	.27	.12	AA	.12	.27	.12	AA	.36	.60	.36

For MDR, we searched for 1-way to 3-way interaction using 10-fold cross-validation. We first looked for the best model among 1-way, 2-way and 3-way interactions in terms of the minimum prediction error. Once the dimension of the interaction model was selected, we then chose the best model with the previously selected order of interaction using the maximum cross-validation consistency. To be comparable with MDR, we allowed up to 3 terms to enter in the model for PLR. The PLR allows up to 3 SNPs to remain in the final model if one specifies the maximum number of terms to be 3. For each simulated dataset, we cross-validated the regularization parameter *λ *for a wide range (1 × 10^-6^, 1 × 10^-4^, 0.01, 1, 100, 100000) according to the BIC criterion [10]. We selected the *λ *that yielded the largest average (cross-validated) log-likelihood and used the chosen *λ *to select a PLR model using again the BIC criterion.

**Figure 2:**
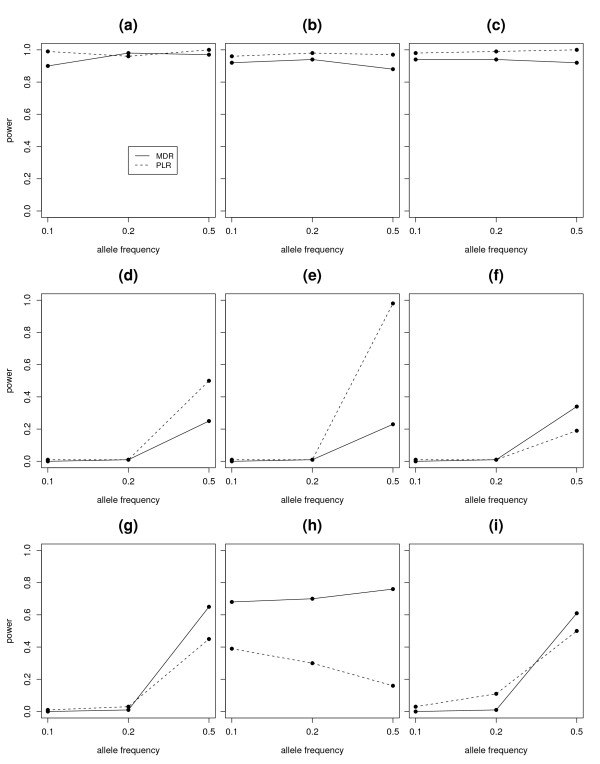
**Power Comparison for two-way interactions**. Figure shows the power of MDR and PLR for detection of interaction under 9 different 2-way interaction models. Eeach plot represents the power of MDR and PLR under different allele frequencies of the associated SNPs. The power of MDR was presented with a solid line and the power of PLR was presented with a dashed line in each figure. The subfigures (a), (b), (c), (d), (e), (f), (g), (h), (i) represent the power plots for Model 1, Model 2, Model 3, Model 4, Model 5, Model 6, Model 7, Model 8 and Model 9 (Table 1) respectively.

We estimated the power of each method as the numbers of times (out of 100) for which the correct SNPs were identified. We also varied the allele frequencies of the associated SNPs to check the impact of allele frequencies on the power of MDR and PLR. Figure 2 summarizes the power comparisons of MDR and PLR when P(A) = P(B) = 0.1, P(A) = P(B) = 0.2, P(A) = P(B) = 0.5 under each of these 9 models in Table 1, where the minor alleles of SNP1 and SNP2 are denoted by A and B respectively. We kept the minor allele frequency of the non-associated SNPs fixed at 0.5.

For the power comparison, we counted the finally selected model as a correct model only if the two SNPs associated with the disease were reported. We did not count a model as a correct model if only one of the two SNPs were reported. So we only counted a model as a correct model for MDR if MDR selected a 2-way model as a best model and the 2 SNPs reported were SNP1 and SNP2. If any SNP other than SNP1 and SNP2 was present in the final model, we did not count it as a correct model. For PLR, we counted a model as a correct model if it reported only SNP1 and SNP2 either as main-effects, or as a two-way interaction. If any SNP other than SNP1 and SNP2 was present in the final model, we did not count it as a correct model for PLR. According to Figure 2, we see that both MDR and PLR had great power to detect interaction under Model 1, Model 2 and Model 3, although PLR had a slightly larger power. Under each of these 3 models, the effect size of at least one of these 9 genotype combinations such as (AA, BB), of the 2 SNPs was hugely different from the other categories. Hence the power to detect association was very good for both these approaches. We noticed that the performance of the MDR approach got worse when P(A) = P(B) = 0.1 under Model 1. This is due to the fact that the case-control ratio became close to 1 at the discriminating combination (AA, BB), when the associated allele frequencies were 10%.

The effect-size difference between the different genotype combinations of SNP1 and SNP2 got substantially smaller in Model 4 as compared to Model 3, even though both were additive models. Both methods lost substantial amount of power in Model 4 compared to Model 3. Still there was some advantage of using PLR as compared to MDR, when the effects were additive (Model 3, Model 4). When the disease allele frequency was 0.5, PLR outperformed MDR under Model 4 and performed even better under Model 5. Model 5 had the same magnitude of main effects for AA and BB genotypes as in Model 4, but had an additional interaction term for the genotype combination (AA, BB) (Figure 1). For Model 5, the expected misclassification error of the one-SNP interaction model is 0.36, which is almost the same as 0.35, the expected misclassification error of the two-SNP interaction model. MDR found it hard to distinguish between these two models. In contrast, PLR was able to still estimate the two main effects separately and sometimes the additional interaction term.

From Model 6 to Model 8, MDR outperformed PLR. Especially in model 8, MDR consistently outperformed PLR for different disease allele frequency. Under model 9, PLR had more power when the disease allele frequency was 0.2 while MDR had more power with allele frequency 0.5. Under model 8 with P(A) = P(B) = 0.5, the expected misclassification errors of one-SNP model and two-SNP model were 0.5 and 0.38 separately, while 0.5 and 0.386 with P(A) = P(B) = 0.1. In both occasions, MDR preferred the two-SNP model over the one-SNP model because of the lower misclassification error rate of the former one. The PLR had a moderate power when P(A) = P(B) = 0.1 and lost power greatly when P(A) = P(B) = 0.5. In the latter situation the expected cases and controls are centered in three genotype cells such as (Aa, Bb), (Aa, BB) and (Aa, bb), while there are very few data points in other cells. This have caused huge instability in the estimation of the parameters, a common problem in parametric approach.

We noticed that the performance of the methods heavily depends on the allele frequency of the associated SNPs. We also noticed that MDR generally outperforms PLR, when the underlying nature of interaction is complex. From the above analysis (Figure 2), we also noticed that when the case/control ratio is about 1, the high-risk or low-risk assignment in the MDR method is unstable. The MDR method also faces the sparseness of data in high dimensions when the allele frequency is way lower than 0.5 (Model 7, Model 9). On the other hand, the estimation of parameters in PLR approach can be heavily affected even when the disease allele frequency is 0.5 (Model 8). We reported the average prediction errors of these two approaches in Table 2. The PLR approach had generally higher prediction errors than the MDR approach, especially when the interaction model was complex. We also checked if the power differences between these two methods were statistically significant. Using a normal approximation to the two binomial distributions, the p-values corresponding to the null hypothesis that the expected probability of rejection is same for MDR and PLR were 0.2446, 0.0317, 0.0115, 0.0005, < 0.0001, 0.0248, 0.0069, < 0.0001 and 0.1548 for Model 1, 2, 3, 4, 5, 6, 7, 8 and 9 respectively for an allele frequency of 0.50.

**Table 2:** Comparison of prediction error between MDR and PLR.

Model	MDR	PLR
Model1	0.077 (0.014)	0.077 (0.013)
Model2	0.131 (0.016)	0.132 (0.015)
Model3	0.123 (0.015)	0.123 (0.015)
Model4	0.379 (0.038)	0.407 (0.047)
Model5	0.331 (0.027)	0.345 (0.026)
Model6	0.413 (0.049)	0.457 (0.062)
Model7	0.383 (0.033)	0.460 (0.065)
Model8	0.386 (0.032)	0.485 (0.038)
Model9	0.382 (0.036)	0.437 (0.048)

The prediction error of MDR and PLR for 2-way interaction models when P(A) = P(B) = 0.5, where A and B are the minor alleles of SNP1 and SNP2 respectively.

### Simulation 2 & Results

**Figure 3:**
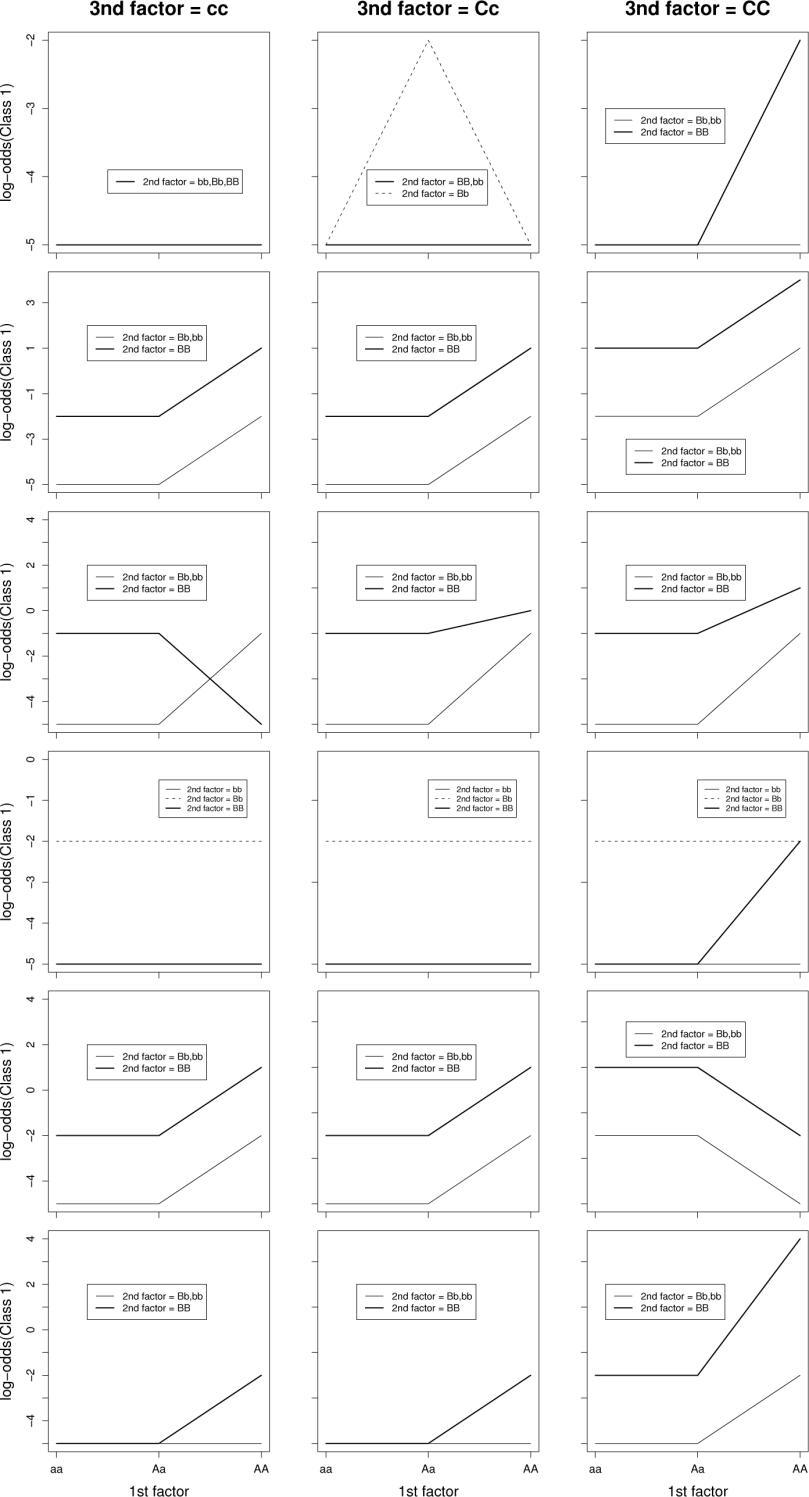
**The pattern of log-odds for 3-way interaction models**. The patterns of log-odds for class1(affected) for different levels of the three SNPs (SNP1, SNP2 and SNP3) under six different models with 3-way interactions. SNP1 has three levels AA, Aa, aa; SNP2 has three different levels BB, Bb, bb and SNP3 has three levels CC, Cc, cc. Each row presents a specific interaction model. The rows 1, 2, 3, 4, 5 and 6 list the interaction models Model 1, Model 2, Model 3, Model 4, Model 5 and Model 6 (Table 3) respectively.

We also compared the power of MDR and PLR for detecting 3-way interactions. We generated 6 epistatic models with different magnitudes of interaction effect. For each model, we simulated 100 datasets. Each dataset contained 400 samples (200 cases and 200 controls) and 20 SNPs, only 3 of which (SNP1, SNP2, SNP3) were associated with the disease. Three levels of the 3 associated SNPs were distributed so that the conditional probabilities of being diseased were as in Table 3. The levels of the remaining 5 SNPs were in Hardy-Weinberg equilibrium for cases and controls, separately. Figure 3 displays the log odds of being diseased for all possible combinations of levels of the 3 associated SNPs. We simulated all the models under different allele frequencies such as 0.1, 0.2 and 0.5 to assess the effect of allele frequency on the power of each approach. We kept the minor allele frequency of the non-associated SNPs fixed at 0.5. We again considered models with just main effects, with only 2nd order or 3rd order interaction effects or with both main and interaction effects to represent the underlying genetic models for the associated SNPs. The coefficients of the models were selected somewhat arbitrarily to cover a wide variety of prevalence and heritability of the disease.

**Table 3:** Penetrance tables: 3-way interactions.

Model 1									
	**3rd factor = cc**			**3rd factor = Cc**			**3rd factor = CC**		

	**Bb**	**Bb**	**BB**	**bb**	**Bb**	**BB**	**bb**	**Bb**	**BB**

aa	0.007	0.007	0.007	0.007	0.007	0.007	0.007	0.007	0.007

Aa	0.007	0.007	0.007	0.007	0.12	0.007	0.007	0.007	0.007

AA	0.007	0.007	0.007	0.007	0.007	0.007	0.007	0.007	0.12

**Model 2**									

	**3rd factor = cc**			**3rd factor = Cc**			**3rd factor = CC**		

	**bb**	**Bb**	**BB**	**bb**	**Bb**	**BB**	**bb**	**Bb**	**BB**

aa	0.007	0.007	0.12	0.007	0.007	0.12	0.12	0.12	0.73

Aa	0.007	0.007	0.12	0.007	0.007	0.12	0.12	0.12	0.73

AA	0.12	0.12	0.73	0.12	0.12	0.73	0.73	0.73	0.98

**Model 3**									

	**3rd factor = cc**			**3rd factor = Cc**			**3rd factor = CC**		

	**bb**	**Bb**	**BB**	**bb**	**Bb**	**BB**	**bb**	**Bb**	**BB**

aa	0.007	0.007	0.27	0.007	0.007	0.27	0.007	0.007	0.27

Aa	0.007	0.007	0.27	0.007	0.007	0.27	0.007	0.007	0.27

AA	0.27	0.27	0.007	0.27	0.27	0.50	0.27	0.27	0.73

**Model 4**									

	**3rd factor = cc**			**3rd factor = Cc**			**3rd factor = CC**		

	**bb**	**Bb**	**BB**	**bb**	**Bb**	**BB**	**bb**	**Bb**	**BB**

aa	0.007	0.007	0.007	0.007	0.007	0.007	0.007	0.007	0.12

Aa	0.007	0.007	0.007	0.007	0.007	0.007	0.007	0.007	0.12

AA	0.007	0.007	0.12	0.007	0.007	0.12	0.12	0.12	0.98

**Model 5**									

	**3rd factor = cc**			**3rd factor = Cc**			**3rd factor = CC**		

	**bb**	**Bb**	**BB**	**bb**	**Bb**	**BB**	**bb**	**Bb**	**BB**

aa	0.007	0.12	0.007	0.007	0.12	0.007	0.007	0.12	0.007

Aa	0.007	0.12	0.007	0.007	0.12	0.007	0.007	0.12	0.007

AA	0.007	0.12	0.007	0.007	0.12	0.007	0.007	0.12	0.12

**Model 6**									

	**3rd factor = cc**			**3rd factor = Cc**			**3rd factor = CC**		

	**bb**	**Bb**	**BB**	**bb**	**Bb**	**BB**	**bb**	**Bb**	**BB**

aa	0.007	0.007	0.12	0.007	0.007	0.12	0.12	0.12	0.73

Aa	0.007	0.007	0.12	0.007	0.007	0.12	0.12	0.12	0.73

AA	0.12	0.12	0.73	0.12	0.12	0.73	0.007	0.007	0.12

Penetrance tables of six different models with 3-way interactions. SNP1 has three genotypes AA, Aa, aa and SNP2 has three different genotypes BB, Bb, bb and SNP 3 has three different genotypes CC, Cc, cc. Each cell of a penetrance table represents the probability of being affected given the cell genotype.

If we denote the associated SNPs by SNP1, SNP2 and SNP3 and the alleles of SNP1, SNP2 and SNP3 by (A, a), (B, b) and (C, c) respectively, then the 6 interaction models were

1. Model 1: Logit(p) = -5 + 3 I(SNP1 = Aa, SNP2 = Bb, SNP3 = Cc) + 3 I(SNP1 = AA, SNP2 = BB, SNP3 = CC)

2. Model 2: Logit(p) = -5 + 3 I(SNP1 = AA) + 3 I(SNP2 = BB) + 3 I(SNP3 = CC)

3. Model 3: Logit(p) = -5 + 4 I(SNP2 = BB) + 4 I(SNP3 = CC) -8 I(SNP1 = AA, SNP2 = BB) + 5 I(SNP1 = Aa, SNP2 = BB, SNP3 = CC) + 6 I(SNP1 = AA, SNP2 = BB, SNP3 = CC)

4. Model 4: Logit(p) = -5 + 3 I(SNP1 = AA, SNP2 = BB) + 3 I(SNP1 = AA, SNP3 = CC) + 3 I(SNP2 = BB, SNP3 = CC)

5. Model 5: Logit(p) = -5 + 3 I(SNP3 = Cc) + 3 I(SNP1 = AA, SNP2 = BB, SNP3 = CC)

6. Model 6: Logit(p) = -5 + 3 I(SNP1 = AA) + 3 I(SNP2 = BB) + 3 I(SNP3 = CC) - 6 I(SNP1 = AA, SNP2 = BB)

The heritability with the minor allele frequency of 0.50 for all SNPs were 7%, 47%, 23%,43%, 5%, and 38% for Model 1, 2, 3, 4, 5 and 6 respectively. Model 1 was a purely epistatic model with no main effect or second order interactions. Model 2 was an additive model with a main effect for each of the 3 SNPs. Model 3 had a main effect term for the 2nd SNP and a third order interaction term. Model 4 was an additive model with pairwise interaction among the 3 SNPs. Model 5 had a main effect term and there was a third order interaction among the SNPs. For Model 6, each of the 3 SNPs had a main effect and a pair-wise interaction term between SNP1 and SNP2 in opposite direction to the main effect. The prevalence of the disease was 0.02, 0.17, 0.13, 0.04, 0.06, 0.12 for Model 1, 2, 3, 4, 5 and 6 respectively.

For MDR, we searched through 1-way to 4-way interaction models using 10-fold cross-validation. With PLR, the maximum number of terms to be allowed to enter into the model was preset at 4. For each simulated dataset, we cross-validated the regularization parameter for a wide range (1 × 10^-6^, 1 × 10^-4^, 0.01, 1, 100, 100000) according to the BIC criterion. We selected the *λ *that yielded the largest average (cross-validated) log-likelihood and used the chosen *λ *to select a PLR model based on the BIC criterion. We counted the finally selected model as a correct model only if the three SNPs associated with the disease were reported. We did not count a model as a correct model if only two of the three SNPs were reported. So we only counted a model as a correct one for MDR if MDR selected a 3-way model as a best model and the 3 SNPs reported were SNP1, SNP2, and SNP3. If any SNP other than SNP1, SNP2, SNP3 was present in the final model, we did not count it as a correct model. For PLR, we counted a model as a correct one if it reported only the SNP1, SNP2 and SNP3 either as main-effects, or as a 2-way or a 3-way interaction. If any SNP other than SNP1, SNP2, SNP3 was present in the final model, we did not count it as a correct model.

**Figure 4:**
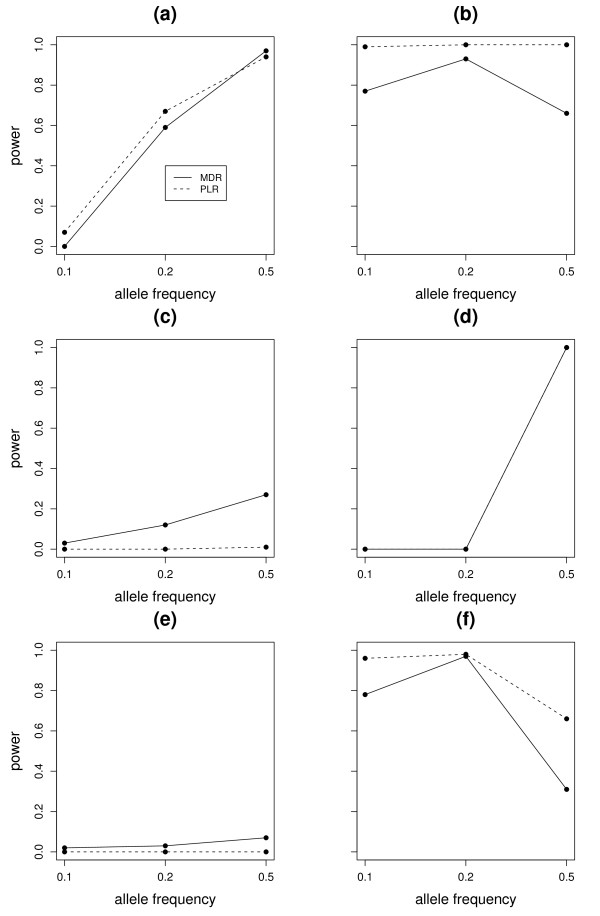
**Power for 3-way interactions**. Figure shows the power of MDR and PLR for detection of interaction under 6 different 3-way interaction models. Eeach plot represents the power of MDR and PLR under different allele frequencies of the associated SNPs. The power of MDR was presented with a solid line and the power of PLR was presented with a dashed line in each figure. The subfigures (a), (b), (c), (d), (e), (f) represent the power plots for Model 1, Model 2, Model 3, Model 4, Model 5, Model 6 (Table 3) respectively.

Figure 4 illustrates the power of MDR and PLR for different allele frequencies of the three associated SNPs separately for each model. We can see that both MDR and PLR had very small power to detect the 3-way interaction when the disease allele frequency was generally low (0.1 or 0.2) under all the models except Model 2 and 6, where the population prevalence of the disease as well the heritability was high. When P(A) = P(B) = P(C) = 0.5, both methods had great power under Model 1 but PLR outperformed MDR under Model 2 and MDR did better under Model 3. We noticed that the power of both methods heavily depends on the allele frequency of the associated SNPs. Moreover, we found that in Model 2, there is definite advantage of using PLR over MDR when the effects are additive (Figure 3). MDR suffered from the sparsity of the cells in higher dimension and equal case-control ratios and hence had lower power than PLR in Model 2, especially when the disease allele frequency was 0.1 or 0.5. On the other hand, when the nature of interaction among the SNPs were complex (Model 3), MDR did a better job than PLR. PLR had almost zero power to detect interaction under all allele frequencies, whereas MDR detected the complex interaction in 25 of these 100 simulations, when the disease allele frequency was 0.5. Both methods perform very similar to detect the pairwise interactions in Model 4 and only detected the interaction model at allele frequency of 0.5. Model 5 had very low population prevalence of the disease and the heritability was quite low. Both approaches did not have adequate power to detect the interaction. For a minor allele frequency of 0.5 for the associated SNPs, the PLR approach had zero power to detect interaction, whereas MDR detected it in 7 of the 100 simulations. Model 6 was very similar to Model 4. It had a 2nd order interaction term in addition to the main effects. The performance of the models were similar to Model 4. The MDR approach performed very similar to PLR for minor allele frequency of 0.2 of the associated SNPs, but it suffered a significant power loss when the minor allele frequencies were 0.1 or 0.5.

We have reported the average prediction errors of these two approaches in Table 4. The PLR approach had generally higher prediction errors than the MDR approach, especially when the interaction model was complex. We also checked if the power differences between these two methods were statistically significant. Using a normal approximation to the two binomial distributions, the p-values corresponding to the null hypothesis that the expected probability of rejection is same for MDR and PLR were 0.4951, < 0.0001, < 0.0001, 1, 0.0209, and < 0.0001 for Model 1, 2, 3, 4, 5 and 6 respectively for an allele frequency of 0.50.

**Table 4:** Comparison of prediction error between MDR and PLR.

Model	MDR	PLR
Model1	0.233(.024)	0.248(0.032)
Model2	0.363(.041)	0.381 (0.043)
Model3	0.185(0.018)	0.197(0.019)
Model4	0.147(0.020)	0.140(0.021)
Model5	0.262(0.019)	0.274(0.021)
Model6	0.228(0.021)	0.235(0.022)

The prediction error of MDR and PLR for 3-way interaction models when P(A) = P(B) = P(C) = 0.5, where A, B and C are the minor alleles of SNP1, SNP2 and SNP3 respectively.

### Real Data Analysis

We have compared the performance of MDR and PLR to detect gene-gene interaction that are associated with acute rejection(AR) in kidney transplant patients. All research subjects reported in the real data analysis were fully consented through a human subjects-approved protocol (IRB Code Number 0006M54661) by the Institutional Review Board of the University of Minnesota in compliance with the Helsinki Declaration. Whole blood was obtained with informed consent and DNA isolated from 271 kidney allograft recipients, 136 of whom had acute rejection (AR) within 6 months of transplant, and 135 of whom did not have any detectable AR after at least 8 years post-transplant. All received Ab induction and CNI, with either MMF or sirolimus. The average age of the rejection group was 44 ± 14.3 years, whereas the age distribution for the non-rejectors was 47.9 ± 12.2 years. There were 56% males and 44% females in the rejection group and the non-rejection group had 58% males and 42% females. The mean time to rejection was 4.8 weeks with a standard deviation (sd) of 7.7 weeks in the rejection group. Among the non-rejectors, the average length of transplant was 12.2 years with a sd of 2.1 years. DNA variants were genotyped using a Affymetrix custom genotyping chip containing 3,590 single nucleotide polymorphisms (SNPs), many of which are thought to be functional variants within biologically relevant genes to acute rejection including genes in pathways associated with immunity, cell signaling, ADME, cell growth and proliferation [19]. Genotyping was performed using the Affymetrix GeneChip Scanner 3000 Targeted Genotyping System (GCS 3000 TG System), which utilizes molecular inversion probes to simultaneously identify the 3404 pre-selected SNPs. Methods for genotyping have been previously described and were performed in strict adherence to the manufacturer's protocol [20]. For this comparison study, we randomly selected 120 caucasian patients with acute rejection within 6 months of transplant, and 120 caucasian patients without any detectable AR after at least 8 years post-transplant.

Of the 3404 SNPs typed, 80 SNPs had no data and hence excluded from the analysis. Of the remaining 3324 SNPs, the call rate was 98.6%. Our goal here was to detect any evidence of interaction among the SNPs associated with acute rejection(AR) in kidney allografts. For all the SNPs, we did Fisher's exact test and selected only the top 100 most significant SNPs for the interaction detection purpose. Among these 100 SNPs, we excluded those SNPs which have minor allele frequency less than 5%. We also excluded those SNPs which have more than 10% missing values. This gave 77 SNPs in the final dataset. We imputed the missing data for each SNP from the observed genotype distribution. Then we applied MDR and PLR methods to detect evidence of interaction among the 77 SNPs and AR.

We reported in Table 5, the top 20 SNPs exhibiting strong association with acute rejection (AR) according to Fisher's exact test. The most significant association was with the SNP rs2147668 in replication factor gene RFC3. The second significant SNP rs875740 was located on the gene ABCC1, which functions as the plasma membrane drug-efflux pump. The third significant one rs2238136 was on the gene VDR, which encodes the nuclear hormone receptor for vitamin D3 and also functions as a receptor for the secondary bile acid lithocholic acid. Some of the other SNPs exhibiting significant associations were involved in pathways affecting drug metabolism or were involved in the immune system. We noticed that two admixture SNPs, which were located in the X Chromosome, also showed significant association. Due to the X Chromosome inheritance, we analyzed these SNPs separately in males and females. We found that both rs2211463 and rs2189394 were highly significant in males, with p values 0.0005 and 0.0004 respectively. But these two SNPs were not significant in females, with p values 0.596 and 0.30 respectively.

**Table 5:** Results from single SNP association analyses: Top 20 SNPs showing significant association with acute rejection according to Fisher's exact test. The p values are not corrected for multiple testing.

SNPs	genes	pvalue	(Putative) Function
rs2147668	RFC3	0.0001	Replication factor C 3
rs875740	ABCC1	0.0006	plasma membrane drug-efflux pump
rs2238136	VDR	0.0009	Vitamin D receptor
rs4988515	IGFBP1	0.0009	Insulin-like growth factor binding protein 1
rs442332	TNFRSF17	0.0009	B-cell maturation factor
rs2211463	Admixture	0.0010	Unknown
rs2189394	Admixture	0.0012	Unknown
rs2741045	UGT1A9	0.0013	Drug metabolizing enzyme
rs288326	FRZB	0.0013	frizzled-related protein
rs1049897	MGP	0.0014	Regulator of cartilage development
rs10276036	ABCB1	0.0015	Initiate T lymphocyte-mediated immunity
rs1800875	CMA1	0.0019	Degradation of the extracelluar matrix
rs4072037	MUC1	0.0021	encodes a mucin glycoprotein(s)
rs11539762	MYEOV	0.0023	myeloma overexpressed gene
rs2241339	ABCB11	0.0024	Progressive intrahepatic cholestasis-2
rs2741046	UGT1A9	0.0028	Drug metabolizing enzyme
rs2014800	ABCC1	0.0030	plasma membrane drug-efflux pump
rs2695232	SOD3	0.0031	superoxide dismutase 3
rs1052369	ANKRD29	0.0033	ankyrin repeat domain 29
rs2305030	LTK	0.0033	leukocyte receptor tyrosine kinase

**Figure 5:**
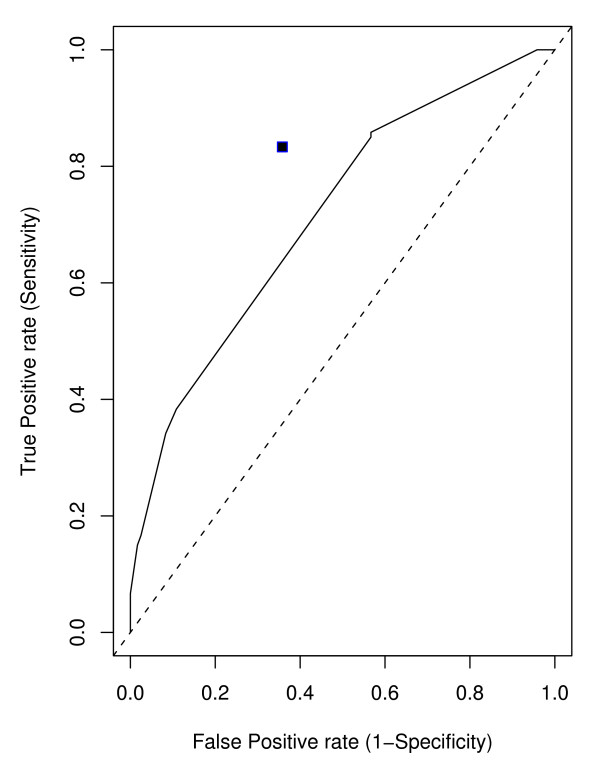
**ROC Curve**. Receiver operating characteristic (ROC) curves for penalized logistic regression. The value for MDR is represented by a solid square in the plot.

**Figure 6:**
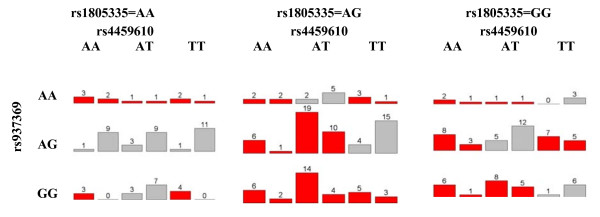
**The case-control distribution for MDR**. The case-control distribution of the finally selected 3-way interaction model for MDR. If a person falls in the red cell, MDR classifies him as a case(AR), otherwise a control.

We first applied MDR on this dataset. We considered only up to 3 way interactions. Ten-fold cross validation was used to obtain the best model for each given number (n = 1,2,3) SNPs. With a given number of SNPs, the training error was used to choose the best model at each cross validation and CV consistency was used to select the best model across the 10-fold cross validation. For one SNP model (main effect model), 6 out of 10 times, MDR chose SNP rs875740 and thus the best model was rs875740. For two SNPs model (2-way interaction model), the best one was rs2741045*rs288326; and for three SNPs model(3-way interaction model), the best model was rs937369*rs4459610*rs1805335. The results of MDR is shown in Table 6. We also reported the averaged training error and averaged test error for these models. We obtained the best overall model based on the averaged test error. The best overall model was (rs937369 (ABCC1)) × (rs4459610 (ACE)) × (rs1805335 (RAD23B)) with averaged test error 0.36 from 10-fold cross-validation. The sensitivity and specificity of the best three SNPs model(also the best overall model)were 0.833 and 0.642 respectively (Figure 5). The p-values from the single SNP association analyses were 0.007, 0.010 and 0.016 for SNPs rs937369, rs4459610 and rs1805335 respectively. Figure 6 shows the distribution of the cases and controls in the 3-way contingency table of the 3 selected SNPs.

**Table 6:** Performance of MDR on kidney acute rejection dataset with 10-fold cross validation

Model	Training Error	Test Error	CV Consistency
rs875740 (ABCC1)	0.37	0.41	6/10
rs2741045*rs288326 (UGT1A9) * (FRZB)	0.32	0.44	4/10
rs937369*rs4459610*rs1805335 (ABCC1) * (ACE) * (RAD23B)	0.25	0.36	4/10

We next fitted a penalized logistic regression model to this kidney AR data. To be comparable with MDR, the maximum number of terms to be entered in the model (also in the selection procedure) is set at 3. Stepwise selection (forward selection and backward deletion) is used to select the final model according to the BIC criterion. Based on the 5-fold cross validated average log-likelihood, we searched the regularization parameter lambda in a wide range [× 10^-6^, 10,000] and ended up with lambda = 0.01 as the optimal tuning regularization parameter. The final model included three main effects rs2147668(RFC3), rs875740 (ABCC1) and rs1138358 (BCL2A1); one 2-way interaction rs2147668 (RFC3) × rs1138358 (BCL2A1). The p-values from the single SNP association analyses were 0.0001, 0.0014 and 0.014 for SNPs rs2147668, rs875740 and rs1138358 respectively.

Penalized logistic regression gives the predicted probabilities of being a case. By changing the cut-off points varying from 0 to 1, we get a serials of sets of sensitivities and specificities. Figure 5 shows the ROC cure for PLR and MDR. The ROC curve by PLR and the solid square by MDR are all away from the identity line y = x towards the upper left corner, indicating that these methods perform far better than random guess. The solid square point is above the ROC curve (Figure 5), suggesting that the overall best model identified by MDR outperformed PLR. Compared to PLR with 0.5 cutoff (Table 7), MDR had higher sensitivity (0.833 vs 0.708) and also had slightly higher specificity (0.642 vs 0.625). MDR also did better than PLR in terms of misclassification error (Table 7). The CV consistencies for MDR were somewhat low. Our sample size was small in terms of detecting interaction. MDR faces from sparseness in high-dimensional contingency table, which probably would have played a role in the low values of cross-validation consistency. The misclassification error, specificity and sensitivity values indicated that the MDR approach provided a reasonable result. We suspect that there are more genes involved that the three genes reported here. Since our sample size was small, we did not feel comfortable to check beyond the third order interaction. We did notice that some other combinations also showed up more than twice in the 10 cross-validation runs for the third order interaction.

**Table 7:** Comparison of prediction performance between MDR and PLR on kidney acute rejection dataset

	Misclassification error	Sensitivity	Specificity
MDR	0.260	0.833	0.642

PLR	0.330	0.708	0.625

The SNP rs937369 is within the first intron of the ATP-binding cassette, sub-family C, member 1 gene (ABCC1). The protein product is a small molecule transporter involved in multi-drug resistance [21]. The protein is ubiquitously expressed throughout the body and has a broad substrate specificity. The SNP rs4459610 results in a nonsynonymous amino acid substitution (p.K715N) in the angiotensin-converting enzyme (ACE). This enzyme catalyzes the conversion of angiotensin I into the physiologically active peptide angiotensin II, which plays a role in the renin-angiotensin system. Variations of ACE have been previously associated with transplant outcome [22]. The SNP rs1805335 is within the fifth intron (IVS5-15A>G) of the human homolog of Saccharomyces cerevisiae Rad23, a protein involved in nucleotide excision repair (NER). RAD23 has recently been implicated in the regulation of targeting protein to the proteasome for degradation [23].

The SNP rs2147668 is within the promoter of the replication factor C (activator 1) 3 gene (RFC3). The protein product is an accessory protein for DNA polymerase delta and epsilon and is involved in sensing DNA damage and DNA replication stress [24]. The SNP rs1138358 is a non-synonymous amino acid substitution (p.K39N) in BCL2-related protein A1 (BCL2A1). This gene is involved in the NF-kappaB signaling pathway resulting in T-cell proliferation in response to inflammatory mediators [25].

Of the genes that contained the identified SNPs, ABCC1, ACE and BCL2A1 have the greatest biological relevancy to transplant outcomes including acute rejection. The protein product of ABCC1 is involved in drug transport and may affect serum concentrations. Variants of ACE have been previously associated with transplant outcomes and BCL2A1 is involved in lymphocyte activation, which would be important in the immune response to a kidney allograft. The protein products of RAD23 and RFC3 are both involved in DNA synthesis and repair with RAD23 also involved in protein degradation. The importance of variants within these genes to transplant outcome is unclear.

## Conclusion

Statistical methods for the detection of gene-gene interactions in a case-control study can be categorized broadly into parametric and nonparametric approaches. PLR is one parametric method which assumes a model to describe the effect of each SNP or combination of SNPs on the disease. MDR is a non-parametric data mining approach which does not make any assumption about the nature of dependence between the trait and the SNPs.

The performance of MDR and PLR are heavily dependent on the allele frequencies of the associated SNPs. Both MDR and PLR work very well when there is a clear pattern in the epistatic model. When there is no such clear pattern, MDR works also well when #case/#control ratio is moderately away from 1 in most (at least half of the cells) genotype combinations. We noticed that due to these issues in high-dimensional contingency table, MDR could not identify all the factors associated with the disease, but correctly identified a subset of the factors. On the other hand, PLR performs better if the nature of the SNP effects is additive, but PLR did not do a great job in capturing complex interaction patterns in our simulation studies. In general, MDR outperformed PLR when the underlined patterns of dependence among the SNPs were complex.

MDR suffers from several technical disadvantages. First, cells in high-dimensional tables will often be empty; these cells cannot be labeled based on the cases/control ratio. Second, the binary assignment (high-risk/low-risk) is highly unstable when the proportions of cases and controls are similar. In high-dimensional interactions, PLR also will have unstable estimates for the parameters and hence will suffer from the loss of power. But, if the SNP effects are really additive, then estimation can be carried out in lower dimensions and hence PLR will have advantage over MDR in this situation.

## Methods

### Multifactor Dimensionality Reduction (MDR)

The MDR method, proposed by [13], is widely used for detecting gene-gene interactions that are associated with common complex genetic diseases. As an alternative to traditional logistic regression, MDR is nonparametric and genetic model free [17] demonstrated that the MDR method identified a four-locus interaction on the risk of sporadic breast cancer and was able to detect a high-order interaction in simulated data in the absence of any statistically significant main effects. Although the MDR method can not distinguish between main effects and interactions, a major strength of the MDR method is its ability to detect higher order interactions even in the absence of main effects.

Let us consider a case-control dataset with N SNPs on n individuals. We have equal number of cases as controls. Suppose M (M ≤ N) is the highest order interaction we want to address. With MDR, multilocus genotypes are pooled into high risk and low risk groups, effectively reducing the dimensionality of the genotype predictors from m dimensions to one dimension [17]. MDR carries out an exhaustive search of all possible 1-way, 2-way, 3-way, up to M-way combinations of predictors (SNPs). The prediction error of each model is estimated using k-fold (usually k = 10) cross-validation. First, we randomly divide the data into k equal parts. The model is developed using each (k-1) parts of the data, the training data, and then used to make predictions about the disease status on the remaining part of the data. The cross-validation is repeated k times and averaged to reduce the bias in the estimation of prediction error.

For any given m (m ≤ M), the general procedure to implement the MDR method to detect the optimum m-way interaction, is illustrated as the following:

1. Run k-fold (often k = 10) cross-validation to find the best set of m-way interactions. For each cross-validation fold, repeat the following steps:

(a) use every possible part as the test data and the other remaining 9 parts as the training data.

(b) a set of m SNPs is then selected from the pool of all SNPs.

(c) m SNPs and their possible multifactor cells are represented in m-dimensional space (The m-way contingency table is formed on the training data)

(d) each multifactor cell is labeled as high-risk if case/control ratio exceeds or equal to some threshold T (eg., T = 1.0), and low-risk otherwise.

(e) MDR searches for all possible combinations of m SNPs. Totally we need to construct () contingency tables and correspondingly get () training errors. The model with the lowest training error (misclassification error) is selected, and the prediction error (test error) of the model is estimated using the independent test data.

2. Calculate the averaged k prediction errors as the prediction error for model size m. Obtain the cross-validation consistency, a measure of the number of times a particular set of SNPs is identified across cross-validations.

We selected the final model size with the lowest prediction error and the final model is selected based on the largest cross-validation consistency. That is, the model that minimizes the prediction error and/or maximizes the cross-validation consistency is selected as the final MDR model.

The goal of MDR is to change the representation of the data using a constructive induction algorithm to make nonadditive interactions easier to detect using any classification method such as naive Bayes or logistic regression [26,27]. This is accomplished by first labeling each genotype combination as high-risk or low-risk using some function of a discrete endpoint such as case-control status. A new MDR variable with two levels is constructed by pooling all high-risk genotype combinations into one group and all low-risk combinations into another group. Evaluation of the predictor can be carried out using cross-validation [28] and permutation testing [29]. Cross-validation is a useful approach for limiting false-positives by assessing the generalizability of models [30].

There is a growing popularity of the MDR approach and it has been recently extensively used for gene-gene interaction detection in many real studies. The strong point in favor of MDR is that it can detect multiple SNPs associated with a disease. It searches through any level of interaction without considering the significance of the main effects. It is therefore able to detect high-order interactions even when the underlying main effects are statistically not significant. On the other hand, MDR suffers from several technical disadvantages. MDR assigns each genotype combination (cell) as high-risk or low-risk and thus converts the high-dimensional dataset to a single dimension. First, cells in high-dimensional tables will often be empty; these cells cannot be labeled as high-risk or low-risk. Second, MDR has a very adhoc way of assigning each cell as high-risk/low-risk. This binary assignment (high-risk/low-risk) is highly unstable when the proportions of cases and controls are similar.

One could calculate the expected prediction error of MDR for an interaction model. For a given interaction model, the penetrance table could be used to calculate the expected number of cases and controls in each cell of a contingency table. If a cell has expected case-control ratio ≥1, the cell is classified as high-risk. Otherwise the cell is classified as low-risk. The expected prediction error is computed as the total number of the controls in all high-risk cells and the number of cases in the low-risk cells divided by the total sample size. For high dimensional interaction, MDR often suffers from the sparsity in high dimensional contingency tables and cannot classify nearly empty cells as high-risk or low-risk accurately. In such cases, a lower dimensional contingency table can provide more information and hence can have lower prediction errors than the model involving true interacting SNPs. In such cases, MDR can only detect a sub-group of the interacting SNPs.

### Penalized logistic regression

The standard logistic regression model has form:

where *X *is a vector of predictors (here genotypes) and *Y *is the affectation status of an individual. The coefficients are typically estimated by maximizing the likelihood, which maximizes the log-likelihood

where *y*_*j *_is the number of affected people at setting *X*_*j *_of a predictor *X*, and *n*_*j *_is the total number of samples at setting *X*_*j*_, j = 1(1)k. [10] proposed using a variant of logistic regression with *L*_2 _regularization to fit gene-gene interaction models. Here the regularization is realized by maximizing the log-likelihood subject to a size constraint on *L*_2 _norm of coefficients and leads to minimizing the following penalized negative log-likelihood:

Here *l*(*β*_0_, *β*) indicates the binomial log-likelihood, and *λ *is a positive constant. The model is fitted by repeating the Newton-Raphson steps which result in the iteratively reweighted ridge regressions (IRRR) algorithm [31].

When the above iteration converges, we get the coefficient estimates. However, none of the coefficients is zero unless the distribution of the factors is extremely sparse. For interpretability, it is necessary to conduct variable selection to include only a subset of predictors in the final model. This is done by forward selection followed by backward deletion. The choice of a factor/interaction to be added in the forward step or deleted in the backward step is based on the score S = deviance + cp × *df*, where cp is complexity parameter and *df *is the effective degrees of freedom. The cp is usually set as 2 or log(sample size) for AIC and BIC respectively. The final model is the one that has minimum score S. The authors claimed that using quadratic regularization with logistic regression has a number of attractive properties. The penalization enables to fit the coefficients in a stable fashion when fitting interactions between categorical factors; Zero cells are handled gracefully.

The obvious advantage of this approach over MDR will be if the real effects are additive. For example, if there are three loci active, and their effect is additive, MDR can only see them all as a three-factor interaction. Typically the power for detecting interactions decreases with number of active loci, since the number of parameters grows exponentially with it. Hence PLR can be a better approach in this case, since the real effects are additive and lower dimensional.

In this paper, we have studied the power of MDR and PLR for detecting gene-gene interaction in a case-control study for various interaction models through simulation studies. We have also studied their performance on a real dataset.

## Competing interests

The authors declare that they have no competing interests.

## Authors' contributions

SB and HH have contributed equally in writing and data analysis. WO and MB have provided the real dataset and were involved in data collection, genotyping of the kidney transplant patients and interpretation of the findings from the dataset. All authors have read and approved the final manuscript.

## Pre-publication history

The pre-publication history for this paper can be accessed here:

http://www.biomedcentral.com/1471-2350/10/127/prepub

## References

[B1] Glazier AM Nadeau JH Aitman TJ Finding Genes That underlie Complex Traits Science 2002 298 2345 2349 10.1126/science.1076641 12493905

[B2] Dreyer SD Zhou G Baldini A Winterpacht A Zabel B Cole W Johnson R Lee B Mutations in LMX1B cause abnormal skeletal patterning and renal dysplasia in nail patella syndrome Nature Genetics 1998 19 47 50 10.1038/ng0598-47 9590287

[B3] Kerem B Rommens J Buchanan J Markiewicz D Cox T Chakravarti A Buchwald M Tsui L Identification of the Cystic-Fibrosis Gene - Genetic Analysis Science 1989 245 1073 1080 10.1126/science.2570460 2570460

[B4] Hollander WF Epistasis and hypostasis J Hered 1955 46 222 225

[B5] Phillips PC The language of gene interaction Genetics 1998 149 1167 1171 9649511 10.1093/genetics/149.3.1167 PMC1460246

[B6] Tyler AL Asselbergs FW Williams SM Moore JH Shadows of complexity: what biological networks reveal about epistasis and pleiotropy Bioessays 2009 31 2 220 227 10.1002/bies.200800022 19204994 PMC3159922

[B7] Bateson W Mendels Principles of Heredity 1909 Cambridge University Press, Cambridge

[B8] Fisher RA The correlation between relatives on the supposition of Mendelian inheritance Trans R Soc Edinb 1918 52 399 433

[B9] Moore JH Williams SM Traversing the conceptual divide between biological and statistical epistasis: systems biology and a more modern synthesis BioEssays 2005 27 637 646 10.1002/bies.20236 15892116

[B10] Park M Hastie T Penalized logistic regression for detecting gene interactions Biostatistics 2008 9 1 30 50 10.1093/biostatistics/kxm010 17429103

[B11] Tibshirani R Regression shrinkage and selection via the Lasso J R Stat Soc 1996 58 267 288

[B12] Kooperberg C Ruczinski I LeBlanc M Hsu L Sequence Analysis using Logic Regression Genet Epidemiol 2001 21 1 S626 S631 11793751 10.1002/gepi.2001.21.s1.s626

[B13] Ritchie MD Hahn LW Roodi N Bailey LR Dupont WD Parl FF Moore JH Multifactor-dimensionality Reduction Reveals HighOrder Interactions among Estrogen-Metabolism Genes in Sporadic Breast Cancer Am J Hum Genet 2001 69 138 147 10.1086/321276 11404819 PMC1226028

[B14] Huang J Lin A Narasimhan B Quertermous T Hsiung C Ho L Grove J Oliver M Ranade K Risch N Tree-structured supervised learning and the genetics of hypertension Proc Natl Acad Sci USA 2004 101 10529 10534 10.1073/pnas.0403794101 15249660 PMC489971

[B15] Nelson MR Kardia SLR Ferrell RE Sing CF A combinatorial partitioning method to identify multilocus genotypic partitions that predict quantitative trait variation Genome Res 2001 11 458 470 10.1101/gr.172901 11230170 PMC311041

[B16] Mandic D Chambers J Recurrent Neural Networks for Prediction: Architectures, Learning algorithms and Stability 2001 Wiley, London full_text

[B17] Ritchie MD Hahn LW Moore JH Power of multifactor dimensionality reduction for detecting gene-gene interactions in the presence of genotyping error, missing data, phenocopy, and genetic heterogeneity Genet Epidemiol 2003 24 150 157 10.1002/gepi.10218 12548676

[B18] Motsinger-Reif AA Reif DM Fanelli TJ Ritchie MD A Comparison of Analytical Methods for Genetic Association Studies Genet Epidemiology 2008 32 767 778 10.1002/gepi.20345 18561203

[B19] Van Ness B Ramos C Haznadar M Hoering A Haessler J Crowley J Jacobus S Oken M Rajkumar V Greipp P Barlogie B Durie B Katz M Atluri G Fang G Gupta R Steinbach M Kumar V Mushlin R Johnson D Morgan G Genomic variation in myeloma: design, content, and initial application of the Bank On A Cure SNP Panel to detect associations with progression-free survival BMC Medicine 2008 6 26 10.1186/1741-7015-6-26 18778477 PMC2553089

[B20] Hardenbol P Baner J Jain M Nilsson M Namsaraev E Karlin-Neumann G Fakhrai-Rad H Ronaghi M Willis T Landegren U Davis R Multiplexed genotyping with sequence-tagged molecular inversion probes Nat Biotechnol 2003 21 6 673 678 10.1038/nbt821 12730666

[B21] Gradhand U Kim R Pharmacogenomics of MRP transporters (ABCC1-5) and BCRP (ABCG2) Drug Metab Rev 2008 40 2 317 354 10.1080/03602530801952617 18464048

[B22] Azarpira N Bagheri M Raisjalali G Aghdaie M Behzadi S Salahi H Rahsaz M Darai M Ashraf M Geramizadeh B Angiotensinogen, angiotensine converting enzyme and plasminogen activator inhibitor-1 gene polymorphism in chronic allograft dysfunction Mol Biol Rep 2008 44 1 86 91 10.1007/s11033-008-9262-z 18454324

[B23] Madura K Rad23 and Rpn10: perennial wallflowers join the melee Trends Biochem Sci 2004 29 12 637 640 10.1016/j.tibs.2004.10.008 15544949

[B24] Zou L Liu D Elledge S Replication protein A-mediated recruitment and activation of Rad17 complexes Proc Natl Acad Sci USA 2003 100 24 13827 13832 10.1073/pnas.2336100100 14605214 PMC283506

[B25] Wang M Windgassen D Papoutsakis E A global transcriptional view of apoptosis in human T-cell activation BMC Med Genomics 2008 23 1 53 10.1186/1755-8794-1-53 PMC2600644 18947405

[B26] Moore JH Gilbert JC Tsai CT Chiang FT Holden T Barney N White BC A flexible computational framework for detecting, characterizing, and interpreting statistical patterns of epistasis in genetic studies of human disease susceptibility J Theor Biol 2006 241 2 252 61 10.1016/j.jtbi.2005.11.036 16457852

[B27] Moore J Dracopoli NC, Haines JL, Korf BR, Moir DT, Morton CC, Seidman CE, Seidman JG, Smith DR Analysis of Gene-Gene Interactions 2008 2 Unit 1.14 Wiley-Liss, Inc., New York

[B28] Hastie T Tibshirani R Friedman J The Elements of Statistical Learning: Data Mining, Inference, and Prediction 2001 Springer, New York

[B29] Good PI Permutation Tests: A Practical Guide to Resampling Methods for Testing Hypothesis 2000 Springer, New York

[B30] Coffey CS Hebert PR Ritchie MD Krumholz HM Morgan TM Gaziano JM Ridker PM Moore JH An application of conditional logistic regression and multifactor dimensionality reduction for detecting genegene interactions on risk of myocardial infarction: the importance of model validation BMC Bioinform 2004 4 49 10.1186/1471-2105-5-49 PMC419697 15119966

[B31] Hastie T Tibshirani R Generalized Additive Models 1999 CHAPMAN & HALL/CRC, Boca Raton 10.1177/096228029500400302 8548102

